# l-Glutamine Attenuates DSS-Induced Colitis via Induction of MAPK Phosphatase-1

**DOI:** 10.3390/nu10030288

**Published:** 2018-03-01

**Authors:** Soo-Yeon Jeong, Yoo Na Im, Ji Young Youm, Hern-Ku Lee, Suhn-Young Im

**Affiliations:** 1 Department of Biological Sciences, College of Natural Sciences, Chonnam National University, Gwangju 61186, Korea; 1606moon@naver.com (S.-Y.J.); youm91@naver.com (J.Y.Y.); 2 Department of Immunology and Institute for Medical Science, Chonbuk National University Medical School, Jeonju 561-180, Korea; yuna@jbnu.ac.kr (Y.N.I.); leeh-k@jbnu.ac.kr (H.-K.L.)

**Keywords:** glutamine, inflammatory bowel disease, DSS, MKP-1, cPLA_2_

## Abstract

Inflammatory bowel disease (IBD), encompassing ulcerative colitis and Crohn’s disease, is a multifactorial inflammatory disease of the small intestine and colon. Many investigators have reported that l-glutamine (Gln) therapy improves outcomes of experimental colitis models, although the mechanism is not fully understood. Regarding the anti-inflammatory properties of Gln, we have shown that Gln can effectively deactivate cytosolic phospholipase A_2_ (cPLA_2_) by rapid induction of MAPK phosphatase (MKP)-1. In this study, we explore the possibility that Gln ameliorates dextran sulfate sodium (DSS)-induced colitis via MKP-1 induction, resulting in inhibition of cPLA_2_, which has been reported to play a key role in the pathogenesis of IBD. Oral Gln intake attenuated DSS-induced colitis. Gln inhibited cPLA_2_ phosphorylation, as well as colonic levels of TNF-α and leukotriene (LT)B_4_. Gln administration resulted in early and enhanced MKP-1 induction. Importantly, MKP-1 small interfering RNA (siRNA), but not control siRNA, significantly abrogated the Gln-mediated (1) induction of MKP-1; (2) attenuation of colitis (colon length, histological abnormality, and inflammation; and (3) inhibition of cPLA_2_ phosphorylation and colonic levels of TNF-α and LTB_4_. These data indicated that Gln ameliorated DSS-induced colitis via MKP-1 induction.

## 1. Introduction

Inflammatory bowel disease (IBD), encompassing ulcerative colitis and Crohn’s disease, is a multifactorial inflammatory disease of the small intestine and colon. Factors implicated in the pathogenesis of IBD include heritable traits, environmental factors, abnormalities in intestinal mucosal barrier integrity and function [1,2], immune regulation [3,4], and gut microbiota [5]. Currently available therapies fail to control symptoms adequately for a significant number of patients, adversely affecting their quality of life [6,7].

Amino acids are key regulators of metabolic pathways, and evidence has indicated additional roles for amino acids in maintaining gut health [8]. The non-essential amino acid l-glutamine, the most abundant amino acid in the bloodstream, is a key respiratory substrate for rapidly dividing cells such as enterocytes [8]. Recent studies have indicated that l-glutamine (Gln) is important for intestinal metabolism, especially following stress [9], and Gln diets improve intestinal morphology and function [10,11,12]. As a result, Gln has long been studied as a promising agent to preserve intestinal function and recovery during injury or stress [13,14]. Many investigators have reported that Gln therapy improves outcomes of experimental colitis models. Although the mechanism by which Gln exerts its beneficial effects is not fully understood, it appears to be correlated with the improvement of barrier function [15], oxidant injury [16,17], and inhibition of inflammatory processes, such as NF-κB activation and TNF-α production [18,19].

Regarding anti-inflammatory properties, we have shown that Gln can effectively deactivate p38 and JNK mitogen-activated protein kinases (MAPKs) by rapid induction of MAPK phosphatase (MKP)-1 [20], which preferentially dephosphorylates p38 and JNK [21,22]. In addition to MAPKs, Gln also deactivates cytosolic phospholipase A_2_ (cPLA_2_) [23,24]. cPLA_2_ hydrolyzes membrane glycerophospholipids at the *sn*-2 position to form arachidonic acid [25]. Arachidonic acid is converted to potent inflammatory lipid mediators, the eicosanoids, which include leukotrienes, lipoxins, thromboxanes, and prostaglandins through the catalytic action of lipoxygenase (LOX) and cyclooxygenase (COX) [26,27]. As a result, cPLA_2_ has been reported to play a key role in the pathogenesis of IBD [28].

Therefore, it is of interest to investigate whether Gln exerts beneficial effects against colitis through MKP-1 induction. In this study, we report that Gln ameliorated dextran sulfate sodium (DSS)-induced colitis via MKP-1 induction, resulting in inhibition of cPLA_2_.

## 2. Materials and Methods

### 2.1. Animals

Specific pathogen-free C57BL/6 mice were obtained from Orient Bio (Seongnam, Gyounggi, Korea) and housed in clean, pathogen-free rooms in an environment with controlled temperature (23 °C), humidity (55%), and a 12 h light/dark cycle. All mice were used at 6–7 weeks of age. All experiments were conducted in accordance with the guidelines of the Chonnam National University Institutional Animal Care and Use Committee (Approval No. CNU IACUC-YB-2015-15). We included 3–5 mice/group/time point/experiment.

### 2.2. Reagents

l-Gln (biotechnology performance certified, G-8540) was purchased from Sigma-Aldrich (St. Louis, MO, USA). Gln was dissolved in sterilized distilled water to reach 4%, the saturated concentration at room temperature, Gln (0.75 g/kg/day) or sham (isovolemic sterile water) was administered to animals by oral gavage. Gln treatment started immediately before DSS treatment was continued throughout the entire 5-day DSS colitis induction. The cPLA_2_ inhibitor pyrrophenone (Cayman Chemical Company, Ann Arbor, MI, USA) was administered (20 mg/kg i.p. in 10% dimethyl sulfoxide) three times (30 min before the initiation of DSS treatment, and days 2 and 4). Control mice received vehicle only. Antibodies against cPLA_2_, phospho-cPLA_2_, and MKP-1 were purchased from Cell Signaling Technology (Danvers, MA, USA).

### 2.3. Colitis Mouse Model

Colitis was induced by administration of 3% DSS (6 < pH < 8; MP Biomedicals, Santa Ana, CA, USA) to the drinking water for 5 days and was replaced daily. Mice were examined daily for symptoms of colitis by monitoring body weight, rectal bleeding, and stool consistency. A disease activity index (DAI) was assigned following the scoring system described by Cooper et al. [29]: (a) weight loss: (0 points: none, 1 point: 1–5%, 2 points: 5–10%, 3 points: 10–15%, and 4 points: >15%); (b) stool consistency/diarrhea: (0 points: normal, 2 points: loose stools, 4 points: watery diarrhea); and (c) bleeding: (0 points: no bleeding, 2 points: slight bleeding, 4 points: gross bleeding). The DAI was calculated as the sum of these scores. The above clinical measures were scored daily throughout the study period. Mice were sacrificed by cervical dislocation.

### 2.4. Histological Examination

Specimens of whole colon without caecum were fixed in formalin and embedded in paraffin blocks. For histological examination, 3-mm paraffin sections were stained with haematoxylin and eosin (H & E). Histological scoring of tissues was performed in a blinded manner by a skilled pathologist as described by Dieleman et al. [30]. Grading index was as follows: inflammation severity (0: none, 1: mild, 2: moderate, 3: severe), inflammation extent (0: none, 1: mucosa, 2: mucosa and submucosa, 3: transmural), crypt damage (0: none, 1: basal one-third damaged, 2: basal two-thirds damaged, 3: only surface epithelium intact, 4: entire crypt and epithelium lost), and the percentage involvement in the ulcer or erosion (1: >1%, 2: 1–15%, 3: 16–30%, 4: 31–45%, 5: 46–100%). The sum of the first 3 scores (inflammation severity, inflammation extent, and crypt damage) was multiplied by the score of the percentage involvement.

### 2.5. Measurement of TNF-α, Leukotriene B_4_

The isolated colons were opened longitudinally and washed with cold PBS, and homogenized in PhosphoSafe Extrction Reagent (Novagen Merck, Darmstadt, Germany) with phenylmethylsulfonyl fluoride protease inhibitor (Sigma-Aldrich, St. Louis, MO, USA). The levels of TNF-α and LTB_4_ were quantified in colonic tissues using an ELISA performed according to the protocol of the manufacturer. The lower limits of detection for the cytokines were as follows: TNF-α (>5.1 pg/mL; R&D Systems, Minneapolis, MN, USA), and LTB_4_ (>13 pg/mL, Cayman Chemical Company, Ann Arbor, MI, USA).

### 2.6. Western Blotting

Specimens of whole colon were collected immediately thereafter, briefly washed with cold phosphate-buffered saline, and dried with blotting paper. The isolated colons were frozen in liquid nitrogen and stored at −70 °C until analysis. Western blotting was performed as described previously [20,23].

### 2.7. Small Interfering RNA (siRNA) Interference

Small interfering RNA (siRNA) strands for MKP-1 and controls were obtained from Santa Cruz Biotechnology (Santa Cruz, CA, USA). In vivo delivery of siRNA was performed using In Vivo-jet polyethyleneimine (PEI; Polyplus-transfection, Illkirch, France), according to the instructions of the manufacturer. In brief, MKP-1 siRNA and PEI dissolved in 5% glucose were mixed in a volume of 200 μL for i.v. injection at room temperature for 20 min, and the mixture was administered 24 h before Gln administration at 2 day intervals. The mixture containing control siRNA and PEI dissolved in 5% glucose without siRNA were used as controls.

### 2.8. Statistical Analysis

Data are expressed as means ± SE. Statistical significance was determined via one-way analysis of variance (Stat-View; Abacus Concepts Inc., Berkeley, CA, USA). A value of *p <* 0.05 was regarded as statistically significant. All experiments were conducted at least twice. Reproducible results were obtained and representative data are, therefore, provided in the figures.

## 3. Results

### 3.1. Oral Gln Intake Improves DSS-Induced Acute Colitis

**Figure 1:**
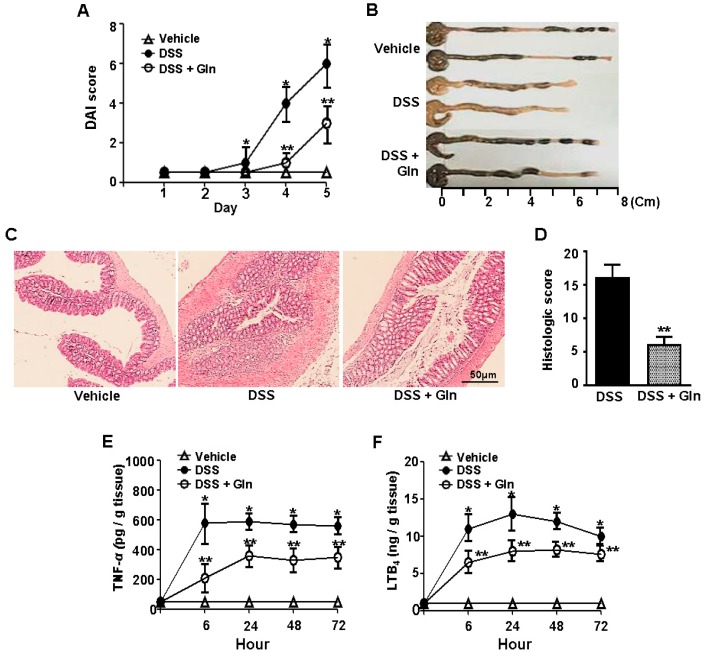
Oral l-glutamine (Gln) intake improves DSS-induced acute colitis. (**A**) Disease severity; (**B**) the colon length on day 5; (**C**) histological examination on day 5; (**D**) histological score; (**E**,**F**) colonic levels of TNF-α and LTB_4_ at 24 h; Data in (**A**,**E**,**F**) represent the means ± SEM of three independent experiments (*n =* 5 mice/group/time point); (**B**–**D**) A representative from three independent experiments (*n =* 3 mice/group/time point) is shown. Scale bar = 50 μm. * *p <* 0.001 vs. vehicle group; ** *p <* 0.05 vs. DSS group in (**A**,**D**–**F**).

In the DSS group, significant weight loss, diarrhea, and bloody stools were observed from day 3. Symptoms gradually increased in the DAI scores that reached a maximum of 5.92 ± 1.21 at day 5. The DAI scores declined thereafter. Bolus Gln treatment significantly improved the colitis-related symptomatology by decreasing the DAI and area under the curve of bleeding and diarrhea scores (Figure 1A). A significant decrease in length of the colons was observed on day 5 in the DSS group. Gln intake significantly restored the reduced colon length (Figure 1B). Histopathological analysis demonstrated that, in contrast to the intact colonic mucosa without histologic alteration in the control group, the DSS group showed disruption of the epithelial barrier with a pronounced decrease in the number of crypts, submucosal edema, and marked infiltration of inflammatory cells into the mucosa and submucosa. Oral Gln intake improved the pathological findings except for mild crypt loss and mild chronic inflammation, as well as a minor extent of affected mucosa and loss of epithelial cells (Figure 1C). Histopathological score revealed that the degree of colitis was significantly lower in the mice treated with Gln than that of DSS control group (Figure 1D).

We examined whether Gln protection from colitis was associated with a decrease in the production of inflammatory molecules. A marked increase in colonic levels of TNF-α (Figure 1E) and LTB_4_ (Figure 1F) at 24 h was observed in the colitis. In contrast, the levels of these molecules were significantly reduced by Gln intake. In this study, we included TNF-α and LTB_4_ as inflammatory mediators. TNF-α is one of the most important pro-inflammatory cytokines. The reason we included LTB_4_ is that we evaluated the degree of cPLA_2_ inhibition by measuring LTB_4_, one of the key cPLA_2_ metabolites. Furthermore, LTB_4_ acts as a potent chemokine by increasing vascular permeability [31,32].

### 3.2. MKP-1 Induction is Crucial for the Gln’s Beneficial Effect

**Figure 2:**
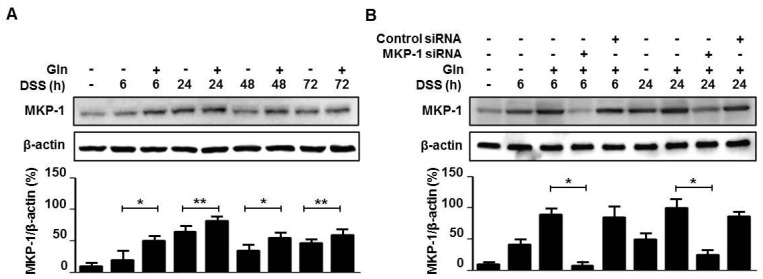
Gln enhances MKP-1 induction in colitis. (**A**,**B**) Immunoblot analysis of MKP-1. Data are representative of three independent experiments (*n =* 3 mice/group/time point). * *p <* 0.05, ** *p <* 0.01 vs. DSS only group in (**A**); * *p <* 0.05 vs. DSS + Gln group in (**B**).

**Figure 3:**
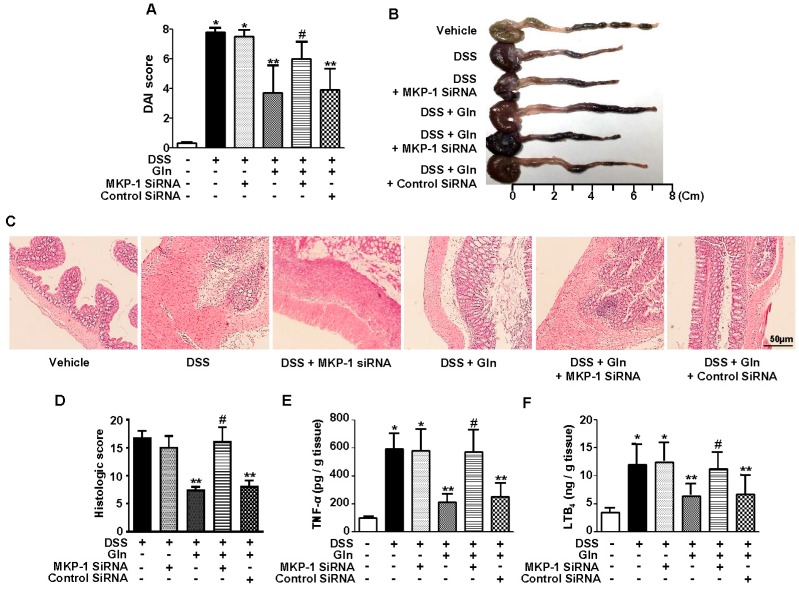
Gln ameliorates colitis via MKP-1 induction. (**A**) Disease severity; (**B**) the colon length on day 5; (**C**) histological examination on day 5; (**D**) histological score; (**E**,**F**) colonic levels of TNF-α and LTB_4_; Data in (**A**,**E**,**F**) represent the means ± SEM of three independent experiments (*n =* 5 mice/group/time point); (**B**,**C**) A representative from three independent experiments (*n =* 3 mice/group/time point) is shown. Scale bar = 50 μm. * *p <* 0.001 vs. vehicle group; ** *p <* 0.05 vs. DSS group; ^#^ *p <* 0.05 vs. DSS + Gln group in (**A**,**D**–**F**).

Western blot analysis revealed that MKP-1 induction was evident in the colon tissue from 6 h and continued to rise over 24 h, after which it declined in response to DSS treatment (Figure 2A). Gln intake resulted in enhanced MKP-1 induction during the entire period of observation from 6 h to 72 h after initiation of DSS treatment (Figure 2A). Mice were administered 0.4 nmol of MKP-1 siRNA or control siRNA via i.v. three times at 2-day intervals beginning 24 h before DSS treatment. MKP-1 siRNA, but not control siRNA, significantly abrogated the Gln-mediated induction of MKP-1 at both 6 h and 24 h (Figure 2B). Importantly, in mice pretreated with MKP-1 siRNA, Gln-mediated attenuation of colitis, i.e., DAI score (Figure 3A), colon length (Figure 3B), and histological inflammation (Figure 3C), and score (Figure 3D), and an increase in colonic levels of TNF-α (Figure 3E) and LTB_4_ (Figure 3F), were no longer observed.

### 3.3. MKP-1 Inhibition of cPLA_2_ Is Associated with the Beneficial Effect of Gln

**Figure 4:**
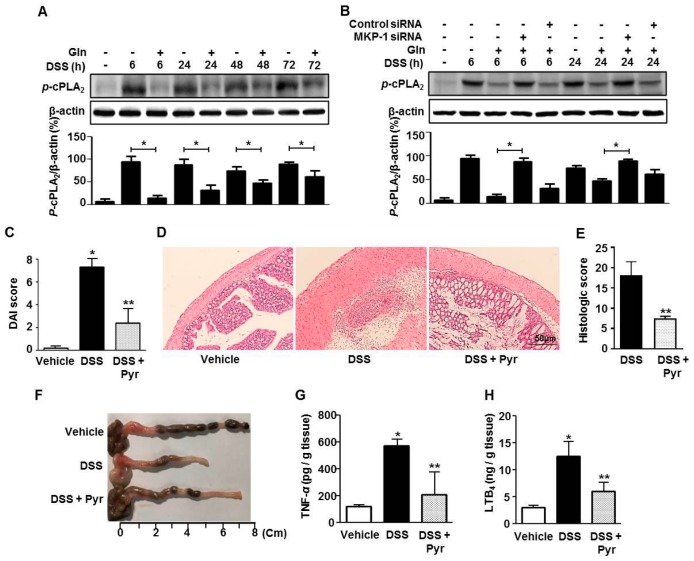
cPLA_2_ inhibition is associated with the beneficial effect of Gln. (**A**,**B**) Immunoblot analysis of cPLA_2_ phosphorylation; (**C**) disease severity; (**D**) histological abnormality; (**E**) histological score; (**F**) colon length; colonic levels of TNF-α (**G**) and LTB_4_ (**H**); (**A**,**B**) A representative of three independent experiments (*n =* 3 mice/group/time point). * *p <* 0.05 vs. DSS only group in (**A**); * *p <* 0.05 vs. DSS + Gln group in (**B**); Data in (**C**,**G**,**H**) represent the means ± SEM of three independent experiments (*n =* 5 mice/group/time point); (**D**–**F**) A representative from three independent experiments (*n =* 3 mice/group/time point) is shown. Scale bar = 50 μm. * *p <* 0.001 vs. vehicle group; ** *p <* 0.05 vs. DSS group in (**C**,**E**,**G**,**H**).

We next examined whether the induction of MKP-1 was associated with cPLA_2_ inhibition in the colons. Strong cPLA_2_ phosphorylation occurred throughout the observation period, and Gln intake inhibited cPLA_2_ phosphorylation regardless of observation time (Figure 4A). MKP-1 siRNA almost completely abrogated the inhibitory effects of Gln on cPLA_2_ phosphorylation (Figure 4B). We finally strengthened the linkage between cPLA_2_ and colitis by examining how cPLA_2_ blockade affects colitis. The cPLA_2_ inhibitor pyrrophenone ameliorated colitis in terms of DAI (Figure 4C), histological inflammation (Figure 4D), and score (Figure 4E), colon length (Figure 4F), and the colonic levels of TNF-α (Figure 4G) and LTB_4_ (Figure 4H). Pretreatment of vehicle-treated normal mice with pyrrophenone did not affect the basal levels of parameters we measured (data not shown), indicating that there were no drug effects in the absence of an insult.

## 4. Discussion

In this study, oral Gln intake was effective in ameliorating DSS-induced colitis such as (1) clinical symptoms evaluated by weight loss, diarrhea, and bloody stool; (2) shortened colon length; (3) histologic abnormality and inflammation; and (4) elevated colonic levels of TNF-α and LTB_4_. Importantly, the present study has demonstrated that Gln ameliorated the colitis in a MKP-1-dependent way. MKP-1 expression was observed during 6–72 h after DSS treatment. Gln administration resulted in earlier and stronger induction of MKP-1 in inflammed colon. MKP-1 siRNA treatment abrogated not only Gln-induced MKP-1 induction, but also all of the Gln’s beneficial effects.

MKPs represent a distinct subfamily within a larger group of dual-specificity protein phosphatases which dephosphorylate MAPK. MKP-1 dephosphorylates and inactivates both p38 and JNK MAPKs, its substrate specificity, and is dependent on cell type and context [21,22,33]. MKP-1 has been reported to be a labile protein that is normally degraded via the ubiquitin/proteasome pathway. Its phosphorylation reduces its ubiquitination and degradation [34,35]. MKP-1 can be rapidly induced in mammalian cells in response to an array of stress stimuli, including oxidative stress and heat shock [36], UV light [37], and DNA-damaging anti-cancer drugs [38,39] through transcriptional [40,41] and post-transcriptional mechanisms [42,43].

**Figure 5:**
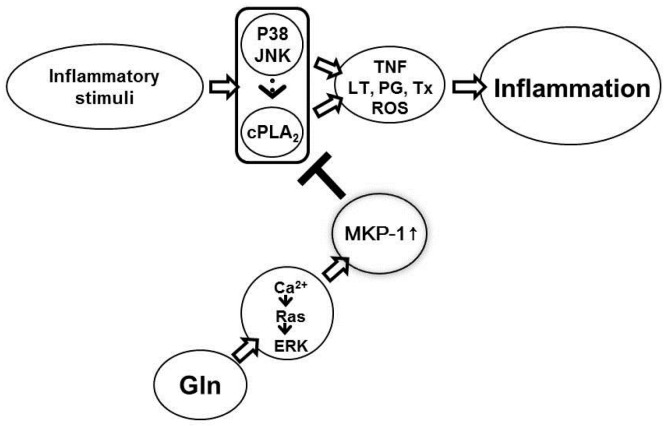
Anti-inflammatory action mechanisms of Gln. Gln binds to G-protein coupled receptors (GPCRs), an allosteric receptor, (unpublished data), and increases ERK activity via activation of the pathway involving Ca^2+^/Ras/c-Raf/MEK (ERK cascade) [41]. ERK phosphorylates MKP-1 on two carboxyl-terminal serine residues—serine 359 and serine 364, which enhances MKP-1 stabilization, resulting in the early induction of MKP-1 [20]. MKP-1 deactivates cPLA_2_ either by dephosphorylating p38 [20,23], which is a major upstream pathway for cPLA_2_ phosphorylation, or by directly dephosphorylating cPLA_2_ due to enhanced physical interaction between Gln-induced MKP-1 and cPLA_2_ [24]. Deactivation of p38 and cPLA_2_ results in suppression of many cardinal inflammatory mediators including reactive oxygen species.

We have shown that MKP-1 induction occurred within 5–6 min following Gln injection in the presence of inflammatory stimuli [20]. This rapid induction suggests that Gln-mediated MKP-1 induction occurs through post-transcriptional mechanisms rather than transcriptional. In this context, Brondello et al. [42] reported that MKP-1 is a target in Vivo and in vitro for ERK, which phosphorylates MKP-1 on two carboxyl-terminal serine residues—serine 359 and serine 364, which enhances MKP-1 stabilization. As the mechanisms of Gln-mediated MKP-1 induction, we have reported that Gln increases ERK activity via activation of the pathway involving Ca^2+^/Ras/c-Raf/MEK [44]. We summarized the proposed anti-inflammatory action mechanisms of Gln in Figure 5.

In this study, cPLA_2_ phosphorylation was observed during the entire period of observation (from 6 to 72 h). Gln intake inhibited cPLA_2_ phosphorylation, as well as the colonic levels of its downstream inflammatory mediator, LTB_4_. MKP-1 siRNA treatment abrogated the Gln inhibition of cPLA_2_ phosphorylation and LTB_4_. cPLA_2_ is involved in a variety of inflammatory diseases through generating eicosanoids, which are potent inflammatory lipid mediators. The involvement of cPLA_2_ in lipid mediator production makes it a potentially important pharmacological target in control of inflammation and cancer. A recent study reported that cPLA_2_ expression and its phosphorylation are increased in the DSS-induced inflammed colon, and cPLA_2_ siRNA ameliorated the colitis [28]. The involvement of COX and LOX in colitis [45,46] further supports an important role for cPLA_2_ in the pathogenesis of IBD. As for the mechanisms of Gln-mediated cPLA_2_ deactivation, we have reported that Gln deactivates cPLA_2_ either by dephosphorylating p38 [20,23], which is a major upstream pathway for cPLA_2_ phosphorylation [47], or by directly dephosphorylating cPLA_2_ due to enhanced physical interaction between Gln-induced MKP-1 and cPLA_2_ [24].

Additionally, many other investigators have reported that Gln therapy improves outcomes of experimental colitis by reducing oxidant injury [16,17], and inhibiting inflammatory processes such as NF-κB activation and TNF-α production [18,19]. It has been reported that cPLA_2_ plays a key role in generation of reactive oxygen species (ROS) through activating NADPH oxidase (NOX) by physical interaction of cPLA_2_ and NOX [48]. Furthermore, cPLA_2_ activation leads to ROS generation. Arachidonic acid, which is formed by cPLA_2_, is converted to eicosanoids through the catalytic action of LOX and COX [26,27]. Both oxygenases have been shown to be capable of producing ROS [49,50,51,52]. In this regard, the ROS-generating ability of cPLA_2_ probably plays a key role in the pathogenesis of IBD, through activation of ROS-sensitive pathways such as NF-κB activation and NF-κB-dependent inflammatory molecules, including TNF-α [53]. Therefore, it is likely that Gln exerts a beneficial effect against colitis by blocking cPLA_2_ via MKP-1 induction.

## 5. Conclusions

We have previously reported that Gln exerts its anti-inflammatory activity via MKP-1-mediated inhibition of cPLA_2_. In this study, we also found a similar mechanism by which Gln exerts strong anti-inflammatory activity against DSS-induced colitis. Therefore, MKP-1-mediated inhibition of cPLA2 appears to be a key mechanism in Gln amelioration of colitis. This study will improve our understanding of how Gln ameliorates IBD.
